# Supplementary material for: Getting More Out of Biomedical Documents with GATE's Full Lifecycle Open Source Text Analytics
Source: PLoS Comput Biol. 2013 Feb 7;9(2):e1002854. doi: 10.1371/journal.pcbi.1002854 (PMC3567135; doi:10.1371/journal.pcbi.1002854)
Supplement: Dataset S1 — GATE software. Dataset S1 bundled with this paper contains a distribution of GATE (or see http://gate.ac.uk/download/). (TGZ) [file pcbi.1002854.s001.tgz › plos-gate/src/gate/resources/gate.ac.uk/tests/html/test2.htm]

The original GATE test document


```
 wsj93_062.0057 
 930119-0125. 
    Diller Is Named Chairman,
@  Chief Executive of QVC :   01/19/93
 WALL STREET JOURNAL (J), PAGE C25 
    QVCN 
 LIMITED PRODUCT SPECIALTY RETAILERS (OTS),
   ALL SPECIALTY RETAILERS (RTS) 
 WEST CHESTER, Pa. 


QVC Network Inc., as expected, named Barry
Diller its chairman and chief executive officer.


Mr. Diller, 50 years old, succeeds Joseph M. Segel, who has been
named to the post of chairman emeritus. Mr. Diller previously was
chairman and chief executive of Fox Inc. and Twentieth Century Fox
Film Corp., both units of News Corp. He also served for 10 years as
chairman and chief executive of Paramount Pictures Corp., a unit of
Paramount Communications Inc.


Arrow Investments Inc., a corporation controlled by Mr. Diller,
in December agreed to purchase $25 million of QVC stock in a
privately negotiated transaction. At that time, it was announced
that Diller was in talks with the company on becoming its chairman
and chief executive upon Mr. Segel's scheduled retirement this
month.
```
